# Biomechanical strategies to maximize gait attractiveness among women

**DOI:** 10.3389/fspor.2023.1091470

**Published:** 2023-02-02

**Authors:** Hiroko Tanabe, Keisuke Fujii, Naotsugu Kaneko, Hikaru Yokoyama, Kimitaka Nakazawa

**Affiliations:** ^1^ Institutes of Innovation for Future Society, Nagoya University, Furo-cho, Chikusa-ku, Nagoya-shi, Japan; ^2^ Graduate School of Informatics, Nagoya University, Furo-cho, Chikusa-ku, Nagoya-shi, Japan; ^3^ Graduate School of Arts and Sciences, The University of Tokyo, 3-8-1 Komaba, Meguro-ku, Tokyo, Japan; ^4^ Institute of Engineering, Tokyo University of Agriculture and Technology, 2-24-16 Nakacho, Koganei-shi, Tokyo, Japan

**Keywords:** locomotion, physical expression, walking, women, social communication, gait, kinematic, gait attractiveness

## Abstract

Physical attractiveness is a key factor in social communication, and through this communication process, we attractively brand and express ourselves. Thus, this study investigated the biomechanical strategies used by women to express gait attractiveness. Our aim was to extend the current literature by examining this aspect of dynamic motion from the perspective of *expressed*, rather than *perceived* attractiveness. In this regard, we obtained motion capture data from 17 women, including seven professional fashion models. The participants walked on a treadmill under two conditions: 1) a normal condition in which they were instructed to walk as casually as possible; and 2) an attractive-conscious condition where they were asked to walk as attractively as possible. Then, we used whole-body kinematic data to represent motion energy at each joint, flexibility of the upper body, and the up-down/forward-backward silhouettes of the limbs, and compared these parameters between the two conditions by using statistical parametric mapping. During the attractive-conscious condition, the non-model women increased the energy of the hip and thoracolumbar joints, which emphasized the motions of their bosoms and buttocks. They also increased their upper body flexibility (possibly reflecting fertility) and continued to face front and downward. Conversely, although the fashion models partially shared the same strategy with the non-models (e.g., hip energy, upper body flexibility, and head bending downward), the strategy of the former was prominent in the stretching of the knee during the push-off phase and pulling the upper arm back, allowing them to showcase their youth and emphasize their chests. In addition, the fashion models used a wider variety of strategies to express their gait attractiveness. The findings indicate that the biomechanical strategy used to express gait attractiveness in women involves showcasing femininity, fertility, and youth. Our results not only deepen the understanding of human movement for self-expression through gait attractiveness, but they also help us comprehend self-branding behavior in human social life.

## Introduction

In social situations, human beings often change their behavior to alter the gaze of other individuals. This communication process is often associated with motion attractiveness, or how a moving person (actor) and his/her observers exchange information related to the actor's attractiveness by using the static and dynamic components of the former's motion. Previous studies have suggested that gait kinematics is a cue for female attractiveness (1, 2), while several researchers have focused on the biomechanical factors of female attractiveness from the observers' viewpoint, and found that the lumbar curvature is an essential static factor for female attractiveness to men (3, 4). Similarly, Morris et al. (5) reported that a shortened stride length and greater hip rotation were key factors for the attractiveness of high-heel walking. Additional contributors to gait attractiveness and an aesthetically optimal gait may include the features of runway walking such as rotating the waist, leaning back, aligning the upper torso, and reducing the arm swing (6). While some studies have examined gait attractiveness from the observer's perspective, few have focused on the actor's perspective and the use of biomechanical strategies to increase perceived attractiveness.

In general, actors communicate their intentions or emotions to observers *via* physical expressions, which consist of whole-body motions or silhouettes. According to Laban Movement Analysis (7, 8), the intentions or emotions hidden in actors' physical expressions are determined by the dynamic features of bodily motions, geometric features of the whole-body shape, the physical body itself, and the destination toward which the body is moving. This approach has been used to create emotional motions in robots by manipulating various parameters, including the energy of each body segment, the matching degree of arm movements (which could be interpreted as the straightness of the motion), and the up-down/retreat-advance silhouettes of the arms (9, 10). These studies in question confirmed that such physical parameters are useful for expressing actors' intentions or emotions.

Therefore, the present study extended these findings by examining the roles of various factors, such as the energy of each body segment, the flexibility of the upper body, and limb silhouettes, in the intentional projection of an attractive female gait. We also investigated the biomechanical strategies used by a sample of professional fashion models and non-models because the former are likely to base their strategies on their previous commercial/cultural training, whereas the latter may be guided by biological/subconscious factors for their perceived attractiveness. Additionally, by investigating the gaits of both groups of women, we focused on the kinematic features for projected attractiveness, as guided by commercial/cultural as well as biological/subconscious factors of motion generation. It is hoped that our findings will not only deepen the understanding of human movement for self-expression through gait attractiveness, but they will also help us comprehend self-branding behavior in human social life.

## Materials and methods

### Ethics

All of the procedures in this study were performed in accordance to the Declaration of Helsinki and approved by the Ethics Committee of the Graduate School of Arts and Sciences at the University of Tokyo. In addition, all of the participants provided their written informed consent to participate in this study and to publish the case details. Informed consent continued throughout the research period *via* dialogs between the researchers and participants.

### Experimental protocols and measurements

A total of 17 healthy women (age: 37.5 ± 8.3 years; height: 165.5 ± 6.5 cm; and weight: 55.1 ± 6.2 kg), including seven professional fashion models (age: 42.4 ± 7.0 years; height: 170.6 ± 3.7 cm; and weight: 55.6 ± 3.4 kg), participated in this study. The models in this study had extensive careers in professional fashion modeling, with a mean duration of 16.6 ± 9.1 years. The remaining 10 participants were members of the general community, three of whom had physical expression experiences such as classical ballet and modern dance (at the amateur level). None of the participants had a significant medical history or signs of gait, postural, or neurological disorders. Although the number of participants in this study was relatively small, the sample size is similar to that of previous research on gait kinematics or kinetics with a within- or between-subjects design (11–14). Moreover, from the statistical analysis perspective, we obtained statistical differences in some gait parameters between the conditions, which raised the possibility of a Type I error.

As for the participants, they were instructed to walk on a treadmill (Bertec, Columbus, OH, United States) with a fixed speed of 1.0 m/s for seven minutes under two conditions: 1) a normal condition in which they were instructed to walk as casually as possible; and 2) an attractive-conscious condition where they were asked to walk as attractively as possible. The order of conditions was counterbalanced. Under the normal condition, the participants were instructed to think about things other than their own walking motion such as food and hobbies. Under the attractive-conscious condition, the participants were instructed to walk as attractively as they could, based on their own perception. It should be noted that the researchers did not present any “correct” gait patterns as part of this process. The participants were allowed to rest for at least five minutes between the trials. A handrail was placed at a height of approximately 1.2 m and a distance of 0.3 m from the participants’ right side, in case of emergency.

Joint motion data was obtained by using a three-dimensional optical motion capture system (OptiTrack V100:R2; NaturalPoint, Corvallis, OR), composed of 12 infrared cameras with a sampling frequency of 100 Hz. Spherical reflective markers (12.7 mm in diameter) were affixed to the following points of each participant's body (42 markers in total): the top of the head, ear, acromion, upper arm, humerus-medial epicondyle (*elbow_in*), humerus-lateral epicondyle (*elbow_out*), wrist, upper margin of sternum (STER), sternum-xiphoid process, lowest edge of rib, C7 vertebra, T8 vertebra, T12 vertebra, anterior superior iliac spine (ASIS), posterior superior iliac spine (PSIS), greater trochanter, femur, lateral knee joint space (*knee_out*), medial knee joint space (*knee_in*), shank, malleolus lateralis (*ankle_out*), malleolus medialis (*ankle_in*), and toe and calcaneus. The *elbow_in*, *knee_in*, and *ankle_in* markers were removed during the walking trials because they interfered with the natural walking movements. Their coordinates were subsequently estimated in an offline analysis.

### Data analysis: motion quality and silhouettes

All of the signal processes were performed using MATLAB R2021a. Since the *elbow_in*, *knee_in*, and *ankle_in* markers were removed during the walking trials, we estimated their positional coordinates using three-point interpolation (15). The joint centers of the ankle, knee, and elbow were calculated as the midpoints between the lateral and medial markers for each joint, while the hip coordinates were calculated using the marker location data for the greater trochanter and ASIS (16). We represented the trunk as a double-link rigid body segmented by the lumbosacral, thoracolumbar, and neck joints. Based on the calculation method developed by (17), we calculated the joint center position of the lumbosacral, thoracolumbar, and neck and shoulder joints by using the ASIS and PSIS (for the lumbosacral joint); the STER, C7, T8, T12, and rib (for the thoracolumbar joint); the STER, C7, and rib (for the neck); and the STER, C7, and acromion (for the shoulder). Then, the time series data for the joint center coordinates were passed through a fourth-order Butterworth low-pass filter (with a cutoff frequency of 6 Hz), using the *filtfilt* function in the MATLAB signal processing toolbox. Additionally, the filtered signals were used for three-dimensional calculations of the joint angles for the ankle, knee, hip, lumbosacral joint, thoracolumbar joint, neck, shoulder, and elbow.

The strides were defined as the beginning and end of the right-heel touching the ground, while foot contact and foot-off time were measured based on the time of the local minimum of the vertical position and the local maximum of the vertical velocity of the heel marker, respectively (18). All of the time series data in this study was normalized to a stride period of 100% and interpolated with 200 sample points to determine the percentage of the stance and swing phases in one gait cycle. The mean values of every minute of the joint angle data were calculated for subsequent data analyses.

As stated earlier, we investigated the biomechanical strategies used by women to increase gait attractiveness. In this regard, we hypothesized that the consciousness of gait attractiveness will result in emphasizing female body parts (i.e., the bosom and buttocks) and showcasing fertility and youth. For this purpose, we focused on the energy of the trunk and hip, the upper body flexibility, and the limb silhouettes resulting from the changes in limb alignment during gait. The energy of trunk and hip joints per unit time was calculated as follows:

$$K_{i}={\left(\frac{d\theta_{i\_sagit}}{dt}\right)}^{2}+{\left(\frac{d\theta_{i\_front}}{dt}\right)}^{2}+{\left(\frac{d\theta_{i\_horiz}}{dt}\right)}^{2},$$

where $\theta_{i\_sagit}$, $\theta_{i\_front}$, and $\theta_{i\_horiz}$ represent each joint in the sagittal, frontal, and horizontal planes, respectively, while *i* represents the hip, lumbosacral, and thoracolumbar joints.

The flexibility of the upper body was calculated as the degree of angular agreement/disagreement between adjacent joints in the sagittal and frontal planes, which is shown as follows:

$$F_{i}=\left|\theta_{i}-{\bar{\theta }}_{i}\right|,$$

where *i* represents the lumbosacral, thoracolumbar, and neck joints; ${\bar{\theta }}_{i}$ is the mean joint angle; and $F_{i}$ of the null represents complete immobility of the joint. Additionally, we investigated the lateral head rotation by calculating $F_{i}$ with $\theta_{neck}$ in the horizontal plane. We also calculated the limb silhouettes in both the upward/downward and forward/backward directions. The upward/downward silhouette was calculated as follows:

$$S\_ud_{i}=r_{i}\cdot \cos\theta_{i},$$

where $\theta_{i}$ represents the joint angle at the left/right knee, hip, shoulder, and elbow in the sagittal and frontal planes, while $r_{i}$ is the segment length of the shank, thigh, upper arm, and forearm, respectively. Moreover, $S\_ud_{i}$ of the positive and negative directions represent the downward and upward deviations of the limb silhouettes.

**Figure 1:**
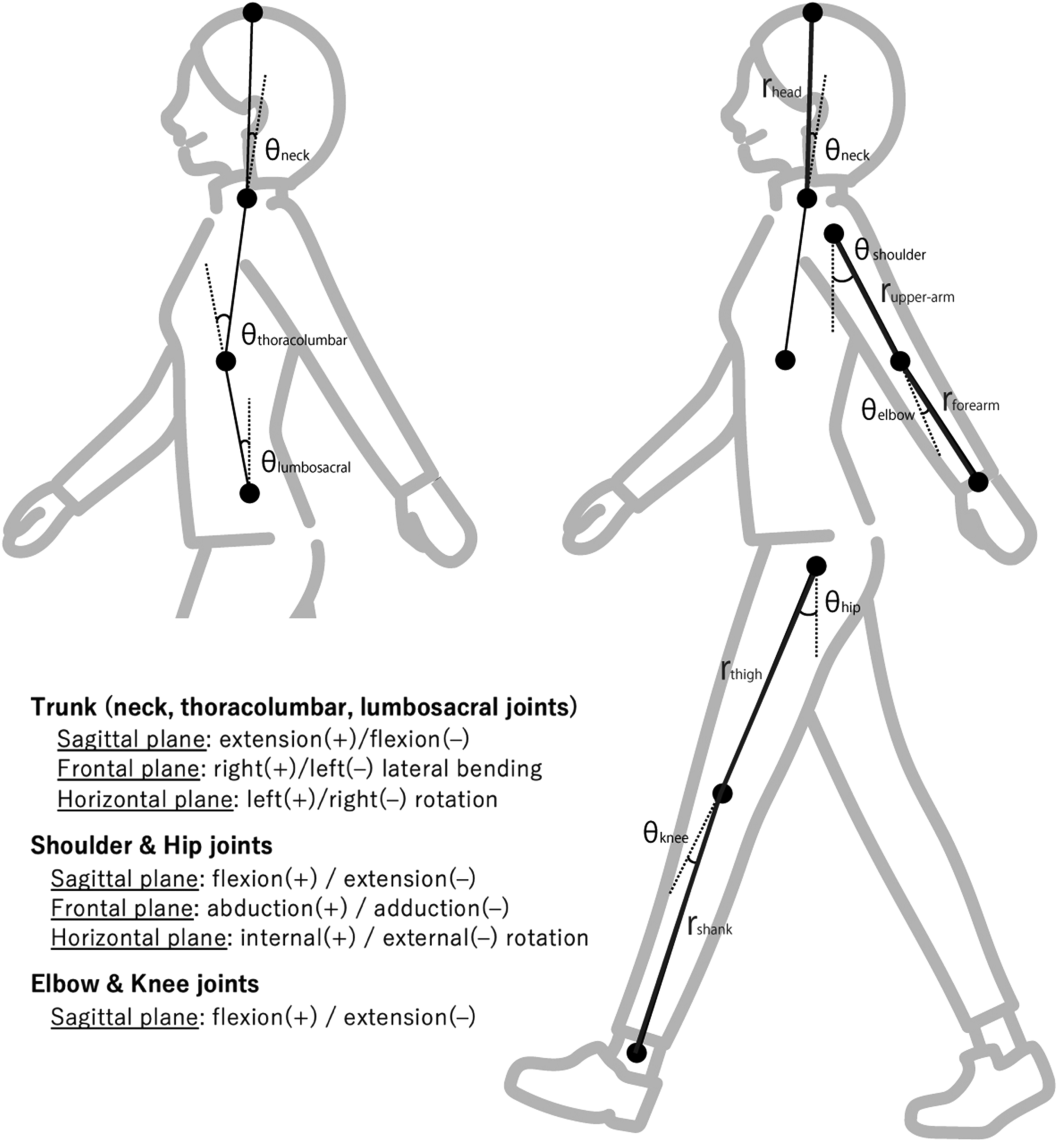
Joint angles and segments used for the calculation of the flexibility (left) and silhouette (right) variables. The definitions of the positive and negative values of each angle are shown in the lower left.

We further investigated the limb and head silhouettes in the forward/backward direction, as follows:

$$S\_f\;b_{i}=r_{i}\cdot \sin\theta_{i},$$

where $\theta_{i}$ represents the joint angle at the left/right knee, hip, shoulder, and elbow, and neck in the sagittal plane, while $r_{i}$ is the segment length of the shank, thigh, upper arm, forearm, and head, respectively. Furthermore, $S\_f\;b_{i}$ of the positive and negative directions represent the backward and forward deviations of the limb silhouettes. Figure 1 shows the joint angle and segment definitions for calculating the flexibility (left figure) and silhouette (right figure) variables.

### Observers' evaluation of gait attractiveness

**Figure 2:**
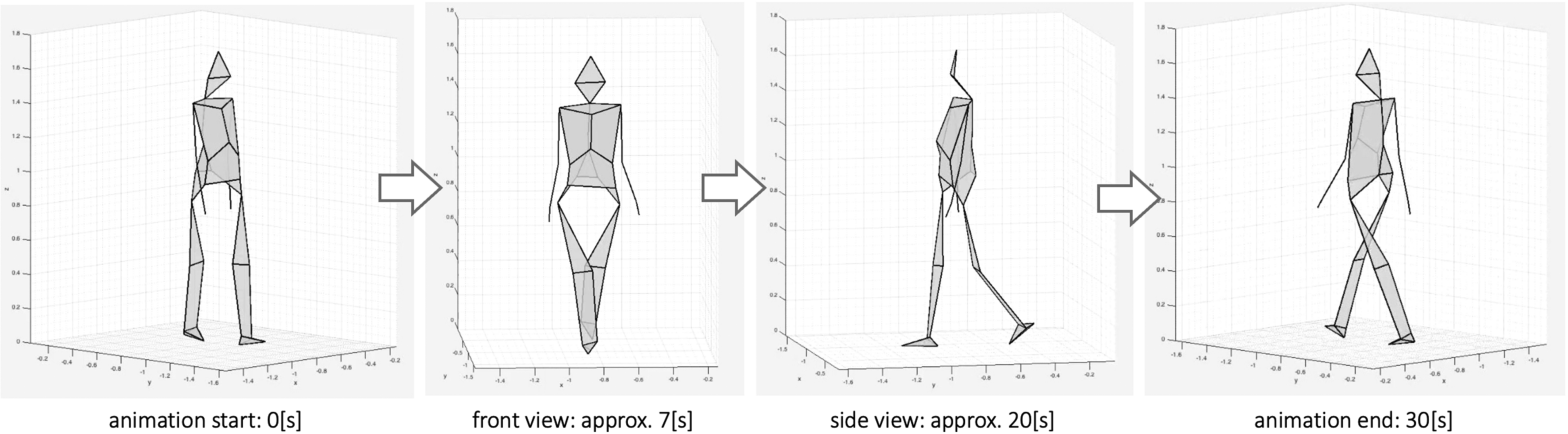
The flow of the 30 second animation presented to the participants in attractive evaluation. Each animation was rotated at a constant speed with a viewpoint from the front right to the back left of the walker.

In order to qualitatively confirm that the walkers' consciousness was transmitted to the observers, we created a 30 s gait animation by using the joint center coordinates of each walker, after which 60 subjects (30 men and 30 women) evaluated the attractiveness of the gaits on a seven-point Likert scale, ranging from 1 (lowest attractiveness) to 7 (highest attractiveness). To create each animation, we used the motion data in 1- to 1.5 minute intervals from the start of the measurement. During the 30 s, we rotated the viewpoint of the animation at a constant speed from the front right to the back left of the walker (Figure 2 illustrates the beginning and end of the animation). All 60 participants viewed the animation, which was presented in randomized order.

### Statistical analysis

The time series data (% gait cycle) in this study was expressed as means ± standard deviations, while the main effect of attractive consciousness was tested using a two-way analysis of variance. The statistical difference at each time (% gait cycle) between the two groups under the two conditions was determined by using statistical parametric mapping (SPM), which was originally developed for analyzing the differences in brain activity data (e.g., fMRI, PET, SPECT, EEG, and MEG time series) and has been recently used for comparisons of time series data for kinematic variables (19, 20). The SPM was calculated using the SPM1D MATLAB package (www.spm1d.org). Moreover, for each variable, a paired t-test, i.e., the SPM{t} function with alpha = 0.05, was computed to analyze the similarity between the conditions. Then, we extracted the time (% gait cycle) when SPM{t} deviated from the assumed threshold, representing the statistical difference between the conditions. Since the focus of this study was the changes in kinematics due to the awareness of self-attractiveness (i.e., the differences between the conditions), we separately analyzed each group.

## Results

### Observers' evaluation of gait attractiveness

To qualitatively confirm that the walkers’ consciousness was transmitted to the observers, 60 subjects evaluated the attractiveness of the gaits on a seven-point Likert scale, ranging from 1 (lowest attractiveness) to 7 (highest attractiveness. For both groups, an attractive-conscious gait received a statistically high score for attractiveness. Specifically, the scores were 3.09 ± 0.53, 3.50 ± 0.43, 3.77 ± 0.44, and 4.29 ± 0.64 for the non-models under the normal condition, the non-models under the attractive-conscious condition, the models under the normal condition, and the models under the attractive-conscious condition, respectively. In addition, we observed significant differences in the group (F [1,30] = 17.21; *p* < 0.001) and condition (F [1, 30] = 6.81; *p* = 0.0140) factors, while no interaction effects were observed (F [1,30] = 0.1; *p* = 0.7582). We also uploaded the animation of the pedestrian that had the greatest difference between the two conditions, as Supplementary materials. These samples included fashion model animations, with a normal condition score of 4.18 ± 1.31 (Video2.mp4) and an attractive-conscious condition score of 5.07 ± 1.51 (Video1.mp4).

### Energy of motion

**Figure 3:**
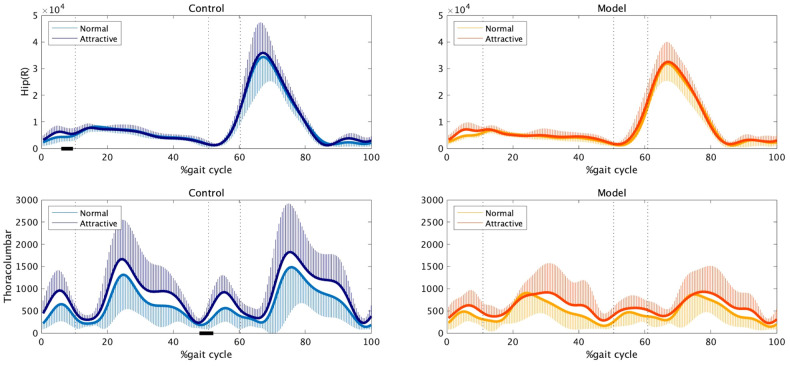
Energy at the hip (top) and thoracolumbar (bottom) joints for the control (left) and model (right) groups. One stride began with a right-heel contact, followed by a left-toe off (approximately 10.3%), a left-heel contact (approximately 50.7%), and a right-toe off (approximately 60.3%), ending with a subsequent heel contact of the same foot. The black bars on the horizontal axis represent significant differences between the two conditions.

In this study, we calculated the energy per unit time in the three-dimensional space at the hip, lumbosacral, and thoracolumbar joints. Among these three joints, the hip and thoracolumbar joints showed statistical differences in energy between the two conditions. Figure 3 presents the average time series of energy during one gait cycle at the hip and thoracolumbar joints, with each group's data plotted separately. The black bars on the horizontal axis represent significant differences between the two conditions.

Under the attractive-conscious condition, the non-models showed greater energy at the hip joint after the ipsilateral foot contact (6%–9% of the gait cycle). Although no statistical difference was observed between the groups, the non-models showed a similar tendency. Additionally, the non-models showed greater energy at the thoracolumbar joints under the attractive-conscious condition, while a statistical difference was observed before and after the onset of the double-stance phase (48%–51% of the gait cycle).

### Upper body flexibility

**Figure 4:**
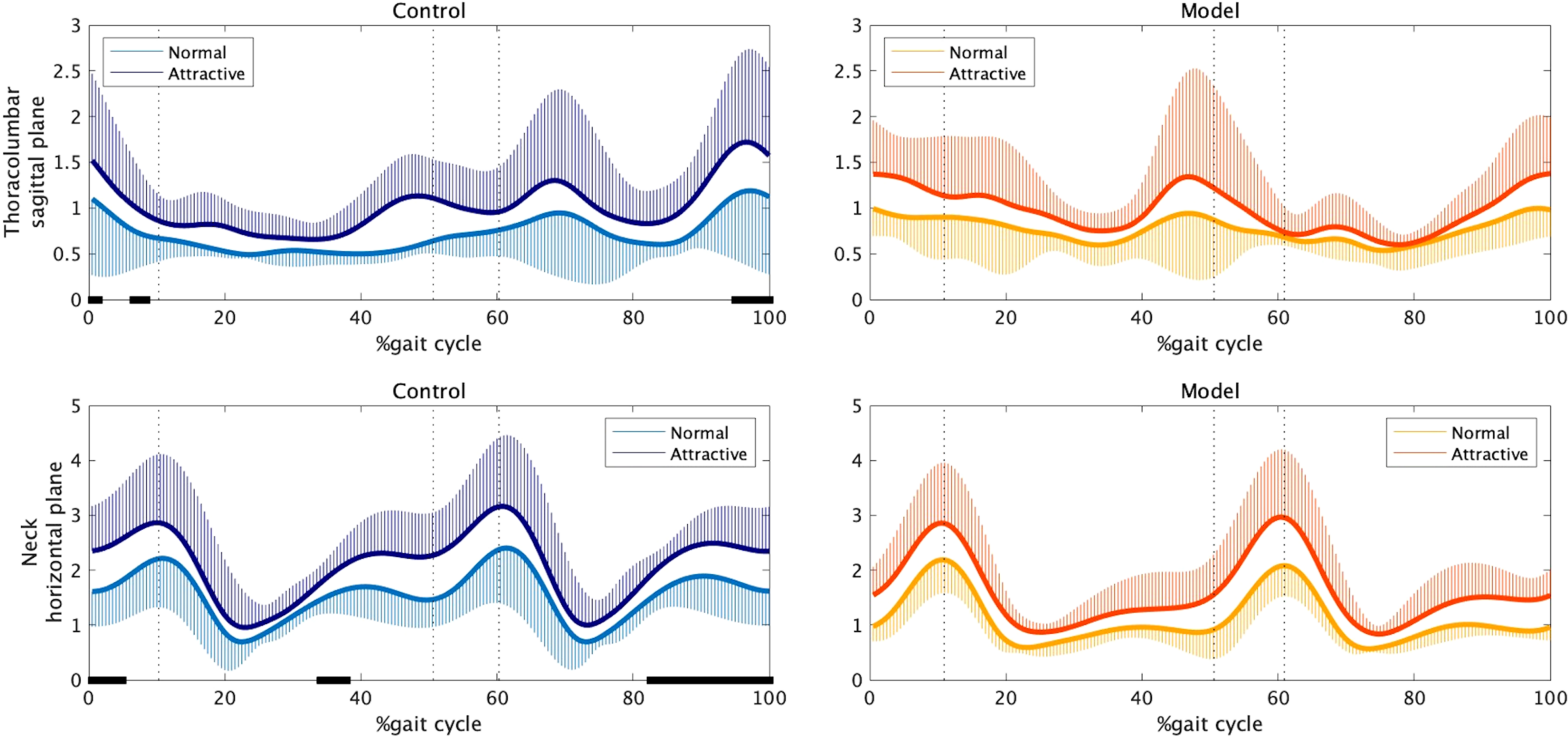
Flexibility at the thoracolumbar joint in the sagittal plane (top) and at the neck in the horizontal plane (bottom). One stride began with a right-heel contact, followed by a left-toe off (approximately 10.3%), a left-heel contact (approximately 50.7%), and a right-toe off (approximately 60.3%), ending with a subsequent heel contact of the same foot. The black bars on the horizontal axis represent significant differences between the two conditions.

The flexibility of the upper body was calculated as the degree of angular agreement/disagreement between adjacent joints in the sagittal and frontal planes. The flexibility at the thoracolumbar joint in the sagittal plane and that at the neck joint in the horizontal plane showed significant differences between the two conditions. Figure 4 presents the average time series of flexibility during one gait cycle at the thoracolumbar joint in the sagittal plane and at the neck joint in the horizontal plane. The flexibility of the non-model group was greater under the attractive-conscious condition than under the normal condition (e.g., the thoracolumbar joint: 0%–1%, 6%–9%, 94%–100% of the gait cycle; the neck joint: 0%–5%, 33%–38%, 82%–100% of the gait cycle). Although similar tendencies were observed for the data of the model group, there was no statistical difference between the conditions.

### Limb and head silhouettes

**Figure 5:**
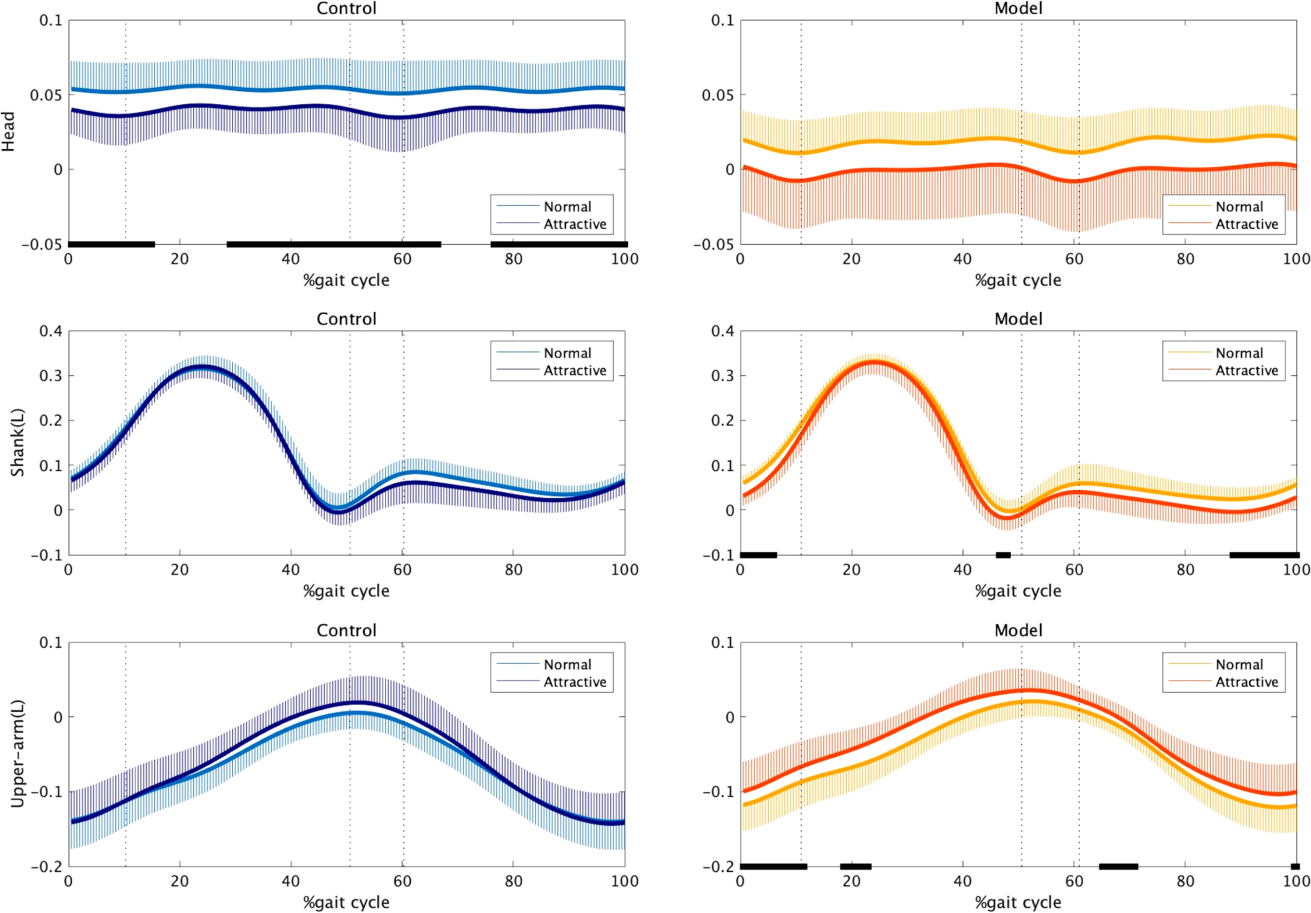
Forward/backward silhouettes of the head (top), shank (middle), and upper arm (bottom). One stride began with a right-heel contact, followed by a left-toe off (approximately 10.3%), a left-heel contact (approximately 50.7%), and a right-toe off (approximately 60.3%), ending with a subsequent heel contact of the same foot. The black bars on the horizontal axis represent significant differences between the two conditions.

We also calculated the limb and head silhouettes as the relative angle between adjacent joints in both the upward/downward and forward/backward directions. The statistical differences in the head and limb silhouettes between the two conditions were only observed in the forward/backward direction. Figure 5 presents the average time series of the forward/backward silhouettes of the head, left shank, and left upper arm. For the non-model group, the attractive-conscious condition was associated with the forward silhouette of the head, which denotes the head bending downward (0%–15%, 29%–66%, and 77%–100% of the gait cycle). Conversely, for the model group, the shank segment under the attractive-conscious condition was located farther forward during the push-off phase, which represents greater stretching of the knees when projecting gait attractiveness (0%–6%, 45.5%–47.5%, and 88%–100% of the gait cycle for the left leg). In addition, the upper arm of the model group showed a backward silhouette, especially during the forward-swing phase under the attractive-conscious condition (0%–11.5%, 18%–23%, 64%–71%, and 99%–100% of the gait cycle).

## Discussion and conclusion

In this study, we investigated the biomechanical strategies used by women to express gait attractiveness by calculating the energy of each body segment, the flexibility of the upper body, and the limb and head silhouettes. Our results showed that the non-models performed an attractive gait by increasing the energy of the hip and thoracolumbar joints, increasing the flexibility of the upper body, and bending the head downward. On the other hand, although the fashion models partially shared the same strategy with the non-models (e.g., hip energy, upper body flexibility, and head bending downward), the strategy of the former was more prominent in the stretching of the knee during the push-off phase and pulling the upper arm back.

Previous studies have indicated that the perception of beauty in human beings is associated with the detection of specific physiological health-related (21–25) and sociocultural (26, 27) features. Mate attractiveness in non-human species is also related to survival and reproduction (28–31). In related research, a female lumbar curvature similar to that observed during pregnancy (approximately 45.5°) has been reported to be the most attractive to men (3) which supports the idea that female attractiveness is related to fertility. Additionally, increased hip rotation and tilt have been associated with attractiveness in female gait (5), suggesting that the motions of sex-specific body parts can be manipulated to alter female attractiveness. In the present study, the women, especially the non-models, showed increased lumbar flexibility (Figure 4) and energy in the bosom and buttocks (Figure 3) when they were conscious of their own attractiveness.

Furthermore, our results showed that the fashion models expressed their attractiveness by altering the backward swing of the upper arm (Figure 5), which emphasized the chest. They also extended the knee during the push-off phase to express attractiveness (Figure 5), which may be considered an expression of the walkers’ health. Regarding the association between health and knee extension, it has been observed in research involving patients with spastic hemiparesis, whose walking capacity is related to the muscle strength of knee extension (32, 33), and in research on aging itself, which demonstrated a correlation between knee extension and age-related declines in the maximum gait speed (34). Our results indicated that the non-models expressed their attractiveness by engaging in movements that showcase their femininity, while the fashion models expressed their health and youth in addition to femininity. This finding suggests that the fashion models tend to use a wider variety of strategies to express their gait attractiveness. This is consistent with the fact that observers' attractiveness judgment of the human body is based on physical features that disclose health and reproductive functions (21–26, 28–31).

In our investigation, the female walkers weakened the link in the horizontal rotation between the head and shoulders (i.e., they tended to face the front) (Figure 4) and tilted the head downward (Figure 5) when walking under the attractiveness-conscious condition. Previous studies have found that head motion is an element that makes up emotional expressions, e.g., keeping the head down when walking is a representation of sadness (35, 36). Meanwhile, facial expressions can convey a similar representation in everyday life (37). However, in the present study, the women attempted to express attractiveness by bending their heads down in a movement that would usually be related to the expression of sadness. Future research is required to determine whether this motion serves any adaptive function, seeing as most behaviors associated with physical attractiveness are hypothesized to reflect adaptations or a necessary byproduct of an adaptation (25). According to the interpretations of bodily movements proposed by Darwin (38) and the Laban Movement Theory (7, 8), the downward bias of the body silhouette represents submission. Again, further consideration of whether showing submission (i.e., the unwillingness to fight) is socially, culturally, and biologically beneficial, as well as perceived as attractive in women, is necessary.

In previous research, it has been reported that walking speed also affects the attractiveness rating (39, 40). Thus, women can vary their walking speed as they express their attraction. However, since walking speed indirectly affects motion, we set the same walking speed for all of the trials in order to purely capture the change in motion, due to the expression of attractiveness. However, this point might be a limitation in our understanding of biomechanical strategy when women express their own gait attractiveness.

In conclusion, the biomechanical strategies used by women to increase the perceived attractiveness of their gait were as follows: increased energy of the hip and thoracolumbar joints; increased curvature of the upper body; pulling back the upper arm; greater knee extension during the push-off phase; and a forward-bending, front-facing movement of the head. These changes in gait conveyed information on the femininity, health, and youth of the women in our sample. Additionally, their expressions of an attractive gait were similar, regardless of their previous backgrounds. Our results can help understand the affective function of the female gait and human movement for self-expression *via* motion attractiveness. In future research, it will be necessary to investigate whether such strategies are correlated with the level of perceived attractiveness reported by observers, which, in turn, can allow us to build a comprehensive model of gait attractiveness from the perspectives of both actors and observers.

## Data Availability

The datasets presented in this study can be found in online repositories. The names of the repository/repositories and accession number(s) can be found in the article/**Supplementary Material**.
